# Case of an Infant with Intermittent Eye Swelling

**DOI:** 10.5811/cpcem.2021.10.54287

**Published:** 2022-01-15

**Authors:** Alexandra H. Baker, Susan Lipsett

**Affiliations:** * Boston Children’s Hospital, Department of Pediatrics/Division of Emergency Medicine, Boston, Massachusetts; † Harvard Medical School, Department of Pediatrics, Boston, Massachusetts

**Keywords:** infantile hemangioma, eye swelling, pediatric emergency medicine

## Abstract

**Case Presentation:**

An eight-week-old infant presented to the emergency department with two weeks of fluctuating swelling and erythema of her right upper eyelid. On examination, she had swelling of the right upper eyelid with ptosis and proptosis as well as a nevus simplex on the upper eyelid. Orbital magnetic resonance imaging demonstrated a proliferating orbital hemangioma.

**Discussion:**

Periorbital erythema and swelling are often infectious or allergic, but in infants with a fluctuating course, underlying vascular malformation must be considered. Without early provider recognition, periocular hemangiomas have the potential to cause vision-related complications.

## CASE PRESENTATION

**Image 1:**
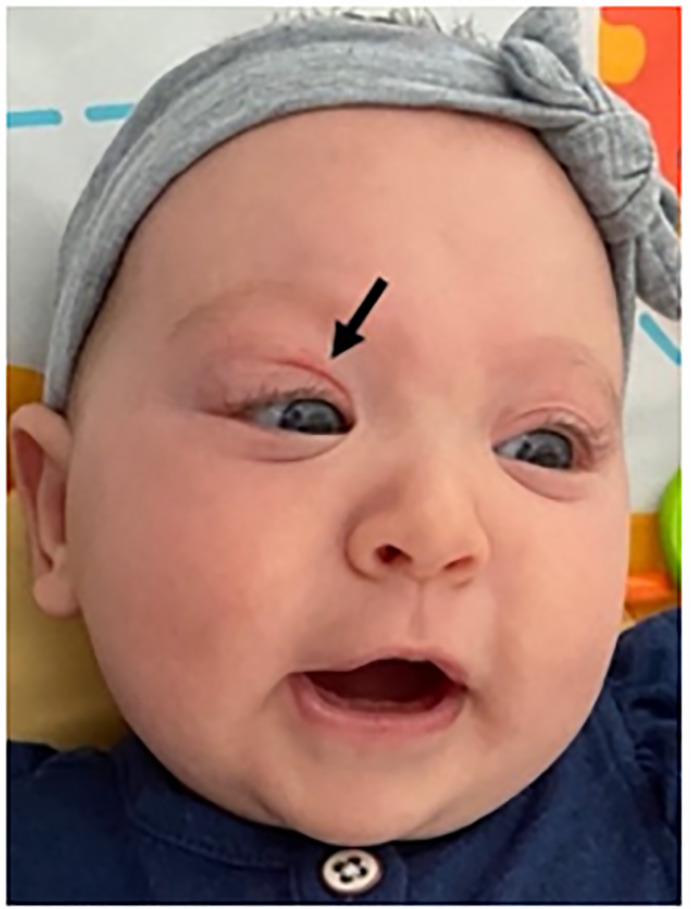
Infant with swelling of the right upper and lower eyelid. Upper eyelid with “angel kiss” (arrow).

An eight-week-old infant presented to the emergency department (ED) with two weeks of fluctuating swelling and erythema of her right upper eyelid. She had been otherwise well without fever, apparent pain, or involvement of the conjunctiva. During her course, she had seen multiple other providers and on day of presentation had been referred from a community ED for concerns of orbital cellulitis. On exam, the patient had moderate swelling of the right upper eyelid with ptosis and proptosis, as well as mild swelling of the lower eyelid. She was also noted to have a nevus simplex “angel kiss” on her right eyelid (Image 1).

**Image 2:**
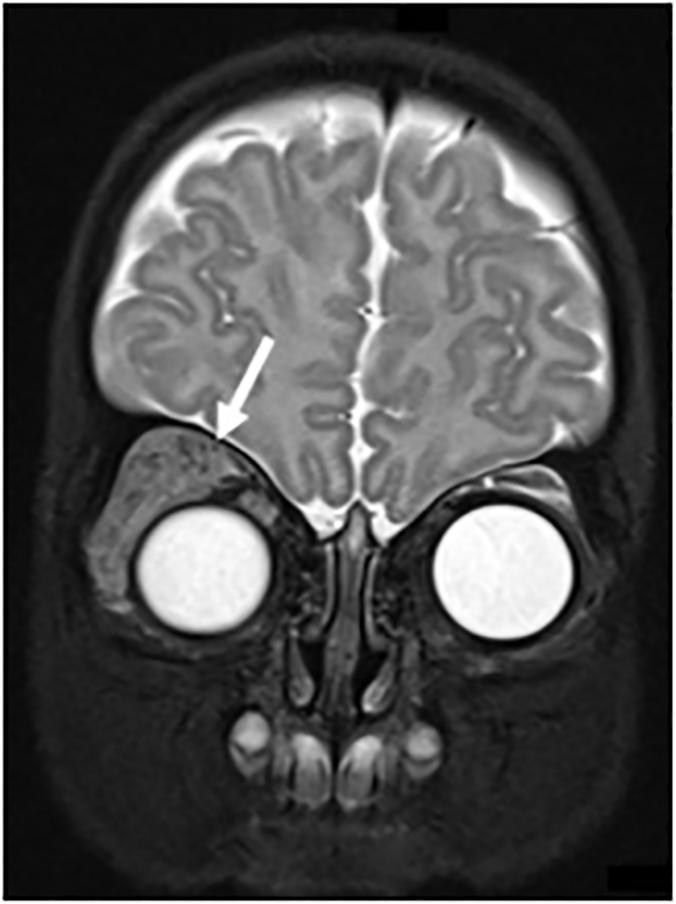
Magnetic resonance imaging of brain and orbits demonstrating signal abnormality consistent with orbital infantile hemangioma (arrow).

Given the fluctuating course of her symptoms and the abnormal exam findings, concern was raised for underlying lesion, and urgent follow-up with ophthalmology was arranged. Magnetic resonance imaging of the brain and orbits revealed signal abnormality in the pre- and post-septal spaces of the right superolateral orbit that involved the right upper eyelid and lateral aspect of the lower eyelid consistent with orbital hemangioma with associated mild, right-sided proptosis (Image 2).

## DISCUSSION

Diagnosis: *Proliferating orbital infantile hemangioma*

Although periorbital swelling and erythema in the pediatric patient are most often consistent with an infectious or allergic etiology, a fluctuating time course and lack of associated symptoms should raise concern for underlying vascular malformation. While a nevus simplex, or “angel kiss,” is often an isolated finding, it can also be associated with deeper vascular lesions and should heighten suspicion. When this is suspected, magnetic resonance imaging and consultation with the appropriate subspeciality can lead to the correct diagnosis and management.

While infantile hemangiomas are the most common benign tumor of infancy and occur in 4–5% of infants,^1^ periocular hemangiomas have the potential to cause vision-related complications. Without physician recognition and appropriate therapy, children are at significant risk for vision loss secondary to amblyopia, astigmatism, strabismus, or corneal exposure and damage related to proptosis.^2^ While difficult to manage surgically due to their location, periocular hemangiomas, like other infantile hemangiomas, generally respond well to medical management.^3^ Our patient was started on propranolol and has had improvement in her swelling. She will continue to be followed closely by an ophthalmologist to monitor her vision development as she ages.

**CPC-EM Capsule**

What do we already know about this clinical entity?

*Infantile hemangiomas are the most common benign tumor of infancy, and periocular hemangiomas have the potential to cause vision-related complications*.

What is the major impact of the image(s)?

*Periorbital erythema and swelling in an infant with a fluctuating course should raise concern for underlying vascular malformation*.

How might this improve emergency medicine practice?

*Emergency physician recognition of a possible underlying vascular lesion will expedite referral to pediatric ophthalmology prior to permanent vision loss*.

## References

[1] Munden A, Butschek R, Tom WL (2014). Prospective study of infantile haemangiomas:
incidence, clinical characteristics and association with placental
anomalies. Br J
Dermatol.

[2] Ceisler EJ, Santos L, Blei F (2004). Periocular hemangiomas: what every physician
should know. Pediatr
Dermatol.

[3] Novoa M, Baselga E, Beltran S (2018). Interventions for infantile haemangiomas of the
skin. Cochrane Database Syst
Rev.

